# Wide-Awake Needle Arthroscopy of the Anterior Ankle: A Standardized Approach

**DOI:** 10.1016/j.eats.2023.102901

**Published:** 2024-01-08

**Authors:** Tobias Stornebrink, Alex B. Walinga, Sjoerd A.S. Stufkens, Gino M.M.J. Kerkhoffs

**Affiliations:** Department of Orthopedic Surgery and Sports Medicine, University of Amsterdam, Amsterdam, The Netherlands; Amsterdam Movement Sciences, Amsterdam, The Netherlands; Amsterdam Collaboration for Health & Safety in Sports (ACHSS), International Olympic Committee (IOC) Research Center Amsterdam UMC, Amsterdam, The Netherlands

## Abstract

Over the years, possibilities in ankle arthroscopy have evolved from diagnostic inspection to complex interventional procedures. Further innovation may come from needle arthroscopy, which has improved substantially in image quality in recent years and can now be used for interventional procedures as well. We here present a standardized approach to wide-awake needle arthroscopy of the anterior ankle under local anesthesia. As new needle arthroscopic procedures of the ankle arise, this approach serves to help ensure safe, uniform, and beneficial adoption of this emergent technique.

Ankle arthroscopy is experiencing increased demand, and over the years, ankle arthroscopic capabilities have evolved from diagnostic inspection to complex reconstructive procedures.^1^ Further innovation may now come from the arthroscopes themselves, as the quality of needle arthroscopy has improved substantially.^2^ Although various needle arthroscopic ankle procedures have been scrutinized, a step-by-step description of a general approach to needle arthroscopy of the ankle is lacking. Such a standardized approach may help to ensure safe, uniform, and beneficial adoption of the technique as new procedures keep emerging.

We here describe a standardized approach to needle arthroscopy of the anterior ankle, which can be performed under local anesthesia.

## Surgical Technique

Video 1 presents the technique in a step-by-step manner. Cadaveric specimens used to display the technique were obtained through the donation program of the Amsterdam UMC university medical center and donated with consent for use in medical science. The study was conducted in agreement with the 1964 Declaration of Helsinki and its later amendments. Ethical approval by our institution’s review board was not required.

### Patient Setup

The patient is positioned in a semi-sitting supine position on a standard operating chair, with the heels hanging just over the edge of the table. Side support can be provided at the pelvic rim contralateral to the operating side. The surgical field is disinfected with a chlorohexidine solution, and standard sterile draping is applied.

### Anesthesia

**Fig 1:**
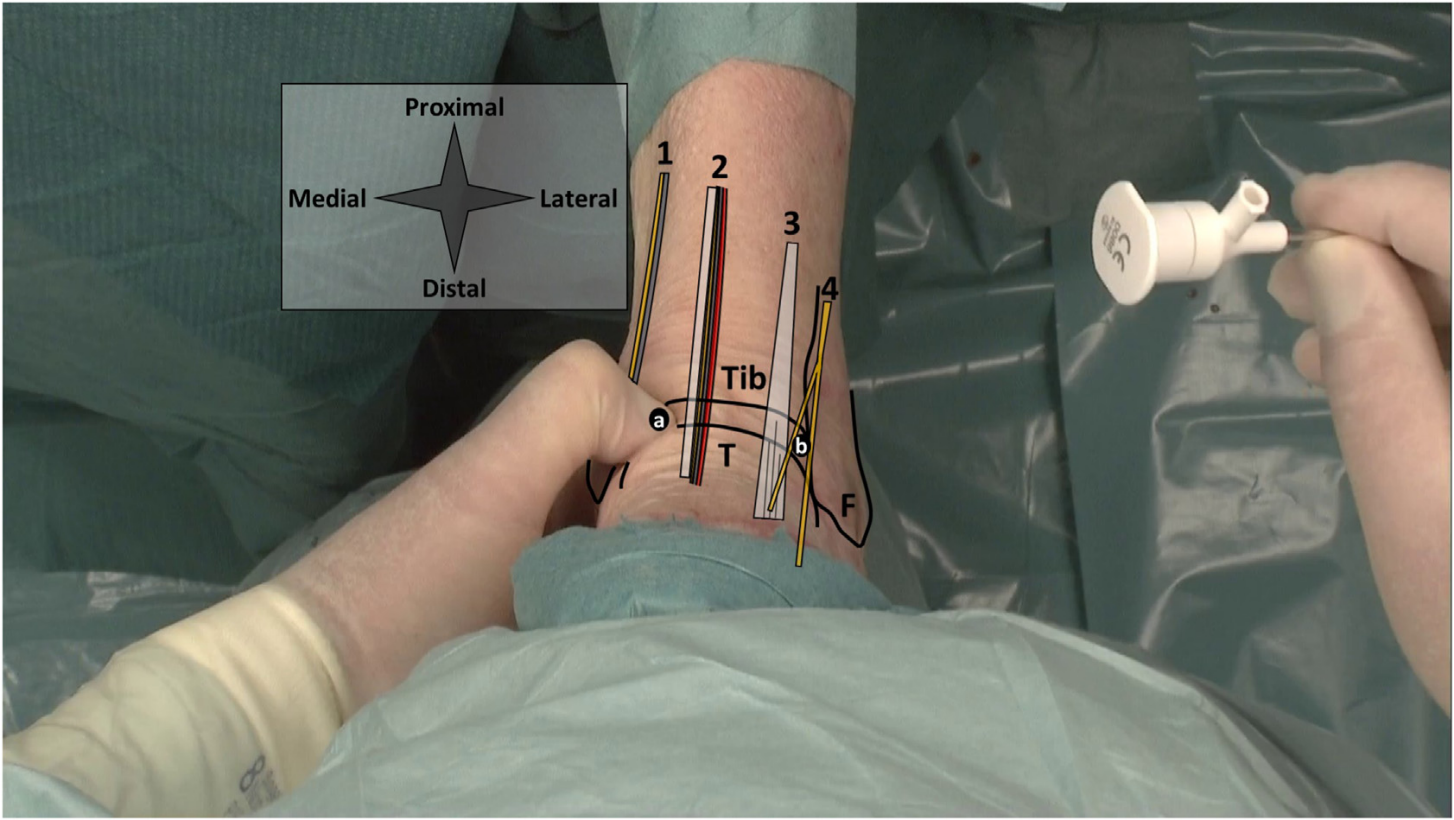
Portal placement in a left ankle, as seen from a top-down surgeon’s perspective with the ankle in supine position. Anatomic landmarks and structures at risk are depicted schematically. The surgeon’s thumb palpates the anteromedial soft spot. The a denotes the anteromedial portal location, and b denotes the anterolateral portal location. From medial to lateral, 1 contains the saphenous nerve and greater saphenous vein, 2 the anterior tibial tendon and anterior neurovascular bundle, 3 the extensor digitorum longus tendon, and 4 the superficial peroneal nerve with its medial dorsal cutaneous branch (superior) and intermediate dorsal cutaneous branch (inferior). (F, fibula; T, talus, Tib, tibia.)

Anteromedial and anterolateral portals are utilized and identified by palpation (Fig 1). With the ankle in dorsiflexion, the anteromedial portal is located on the soft spot, lateral to the saphenous nerve and vein, just medial to the anterior tibial tendon and at the anterior joint line.^3^ The anterolateral portal is located lateral to the peroneus tertius tendon (or to the extensor digitorum longus in case of absence of the tertius), avoiding the frequently visible superficial peroneal nerve, again at the anterior joint line.^3^ Upon localization, the portals are anesthetized with 10 cc lidocaine 2%. The entire portal tract is anesthetized, from skin to joint capsule and intra-articular. The joint capsule should be properly addressed as this is well-innervated tissue. Sedation is not required for this procedure.

### Portal Placement, Arthroscope Introduction, and Joint Distention

**Fig 2:**
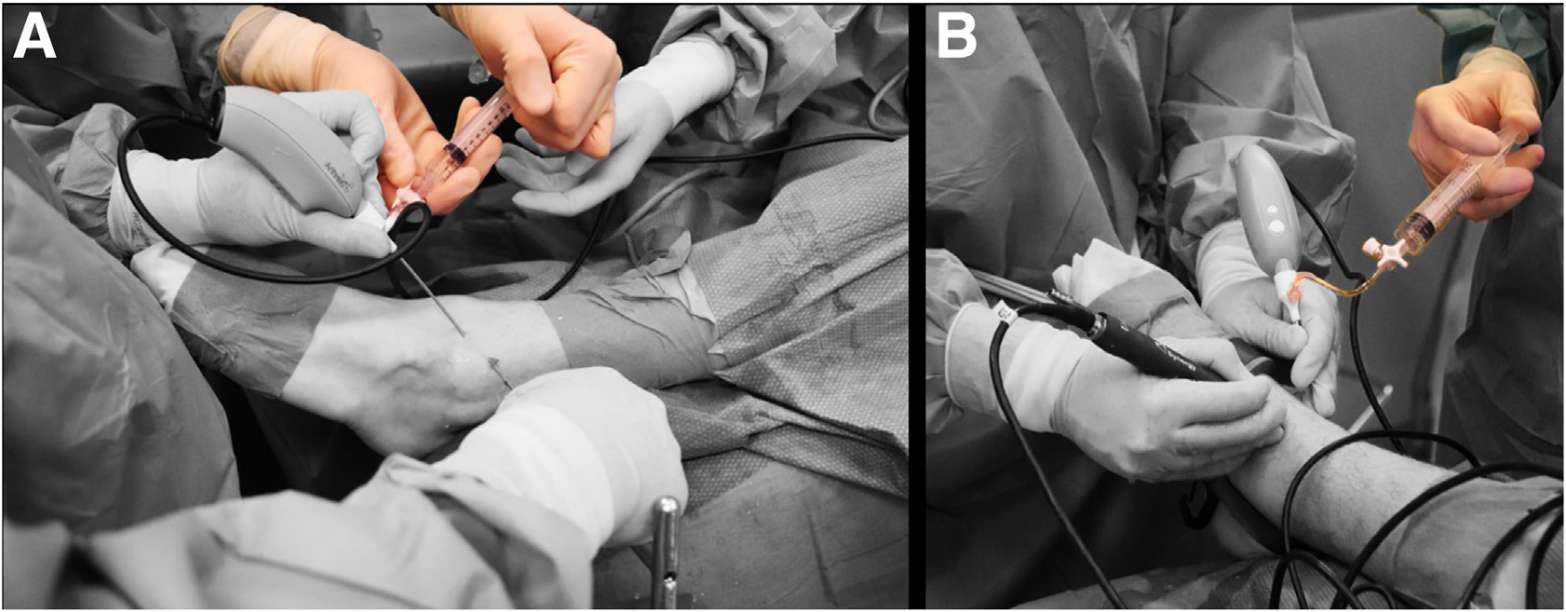
Syringes can be used to distend the joint with saline through the needle arthroscopic viewing portal. Connecting these syringes directly to the needle arthroscopic cannula will interfere with stability, especially during exchange of empty syringes (a). Connecting the syringe through a flexible tube and stop-cock will ensure stability and prevent backflow during exchange of syringes (b).

**Fig 3:**
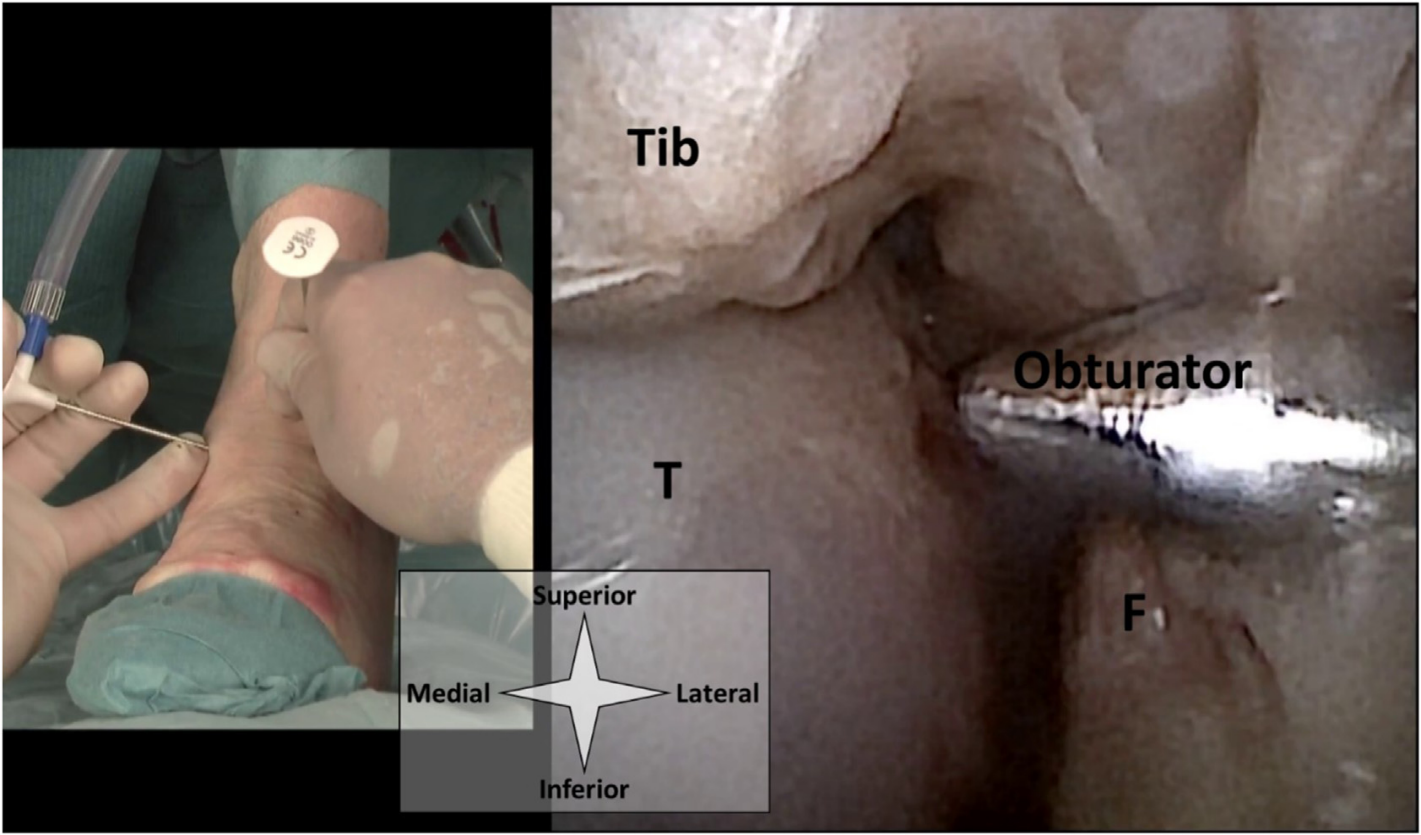
A left ankle from a top-down surgeon’s perspective with the needle arthroscope introduced through the anteromedial portal. The corresponding intra-articular view is shown. The anterolateral portal can be placed guided by intra-articular visualization from the anteromedial portal. Here a blunt obturator is used to mark the anterolateral portal. (F, fibula; T, talus, Tib, tibia.)

The anteromedial portal is created first. The skin is prepared with a 2-mm stab incision by use of a No. 11 surgical blade. A 2.2-mm (for the NanoScope; Athrex) or 2.4-mm (for the NanoNeedle; Athrex) diameter cannula is then loaded with a blunt obturator, and the cannula is penetrated through the joint capsule and entered intra-articular. During portal placement, the ankle is maintained in dorsiflexion to protect the talar weightbearing cartilage. Slight noninvasive distraction may be of help in achieving intra-articular positioning. The obturator is removed from the cannula and replaced with the 1.9-mm diameter needle arthroscope (NanoScope or NanoNeedle). This needle arthroscope is semi-rigid and has a 0° direction of view. The joint can now be distended by connecting a source of sterile saline to the cannula. We use either 1 of 3 sources for distention: a syringe, a pressure IV, or an arthroscopic pump. When using a syringe, stability of the arthroscope is increased by connecting the syringe through a flexible tube and stop-cock (Fig 2). The anterolateral portal can now be established under intra-articular visualization (Fig 3). Proper positioning is first confirmed with a 21-gauge (green) needle. Further steps are equal to those described for the anteromedial portal, again using a 2-mm stab incision of the skin and a cannula loaded with a blunt obturator to penetrate the joint capsule.

### Inspection

**Fig 4:**
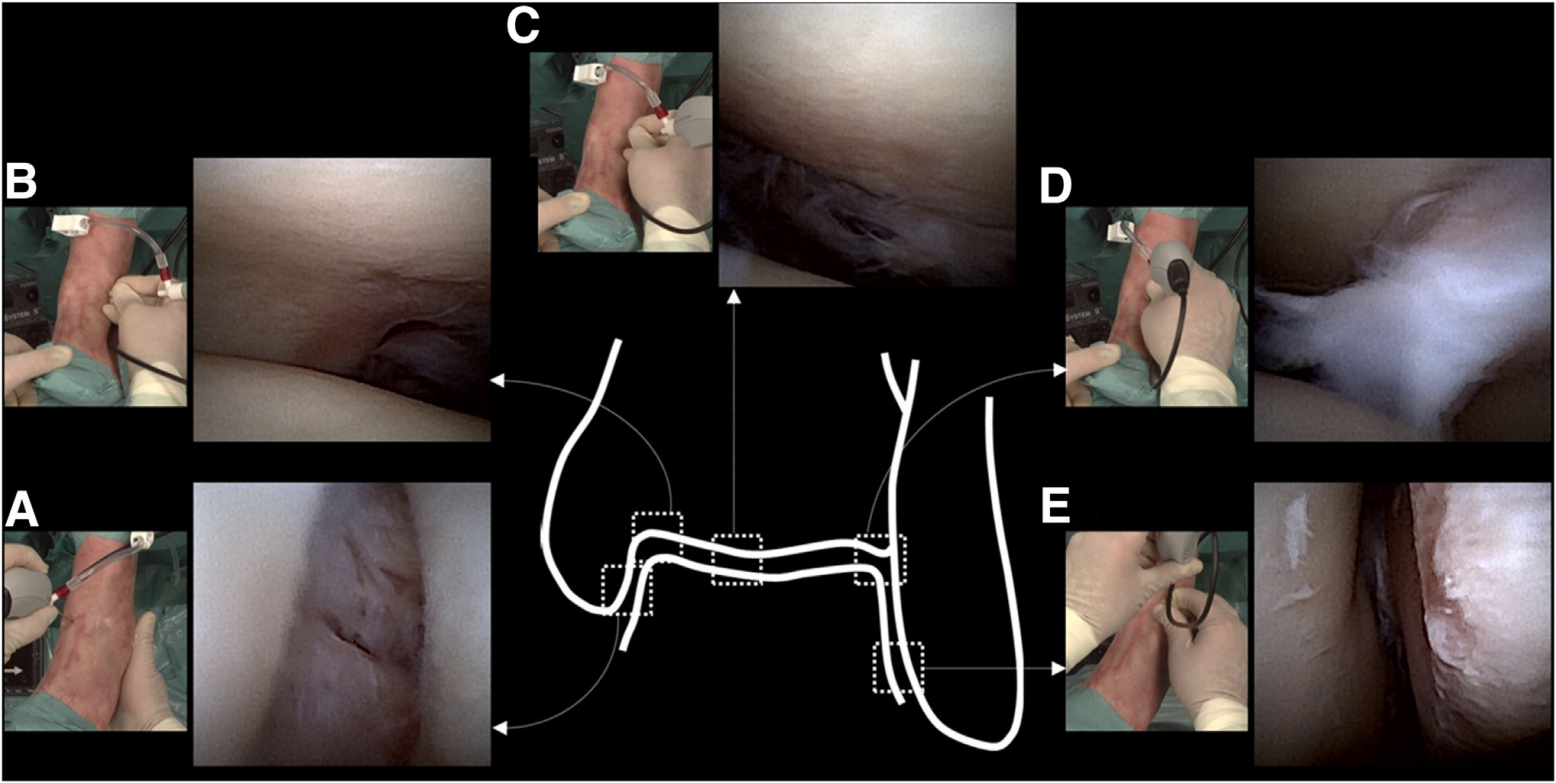
Inspection of a left ankle. The schematic drawing shows the approximate location of the corresponding needle arthroscopic images. Each needle arthroscopic image is accompanied by a photo of the needle arthroscope from a top-down surgeon’s perspective, at the exact moment of inspection. The anteromedial portal is used for inspection of the deltoid ligament and medial gutter (a). The anterolateral portal is used for inspection of the medial talus and tibia (b), central and posterior talus and tibia (c), talofibular articulation (d), and the lateral gutter (e).

**Fig 5:**
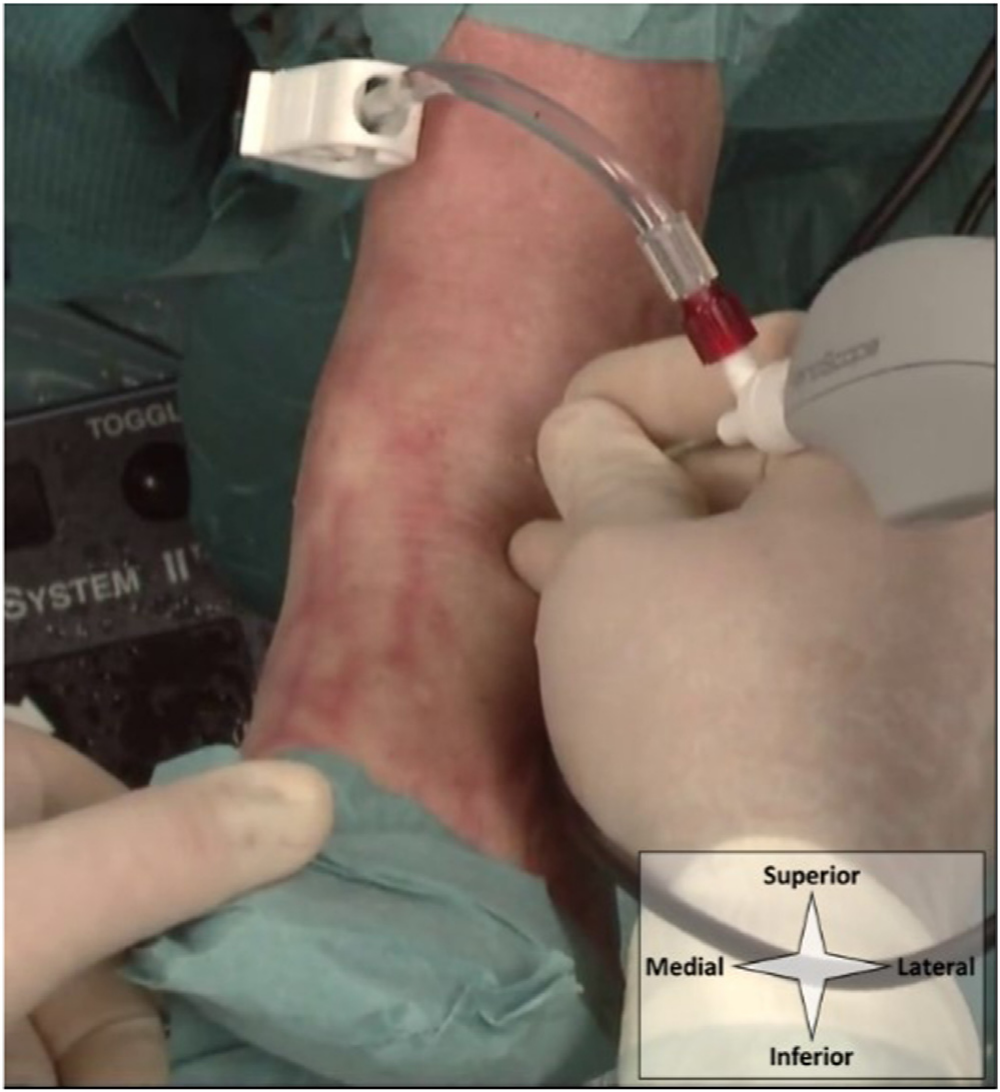
A left ankle in supine position seen from a top-down surgeon’s view. The needle arthroscope is inserted in the ankle joint through the anterolateral portal and held with a pencil grip.

A thorough inspection of the ankle joint can now performed according to Vega et al.^4^ Starting in the medial gutter, we work through the joint from medial to lateral and from superior to inferior and make sure to properly inspect the deltoid ligament, medial gutter, medial/central/lateral talus and tibial plafond, talofibular articulation, lateral gutter, and anterior gutter (Fig 4). The arthroscope may be alternated to the anterolateral portal to aid in inspection, and a probe can be inserted in the contralateral portal as well. Holding the arthroscope distally on its camera tube and with a pencil grip will aid in maintaining stability with this delicate equipment (Fig 5). As the arthroscope and its cannula have a semi-rigid frame that can be bent to some extent, they may be carefully moved through the joint to inspect more posteriorly.

### Biopsies

**Fig 6:**
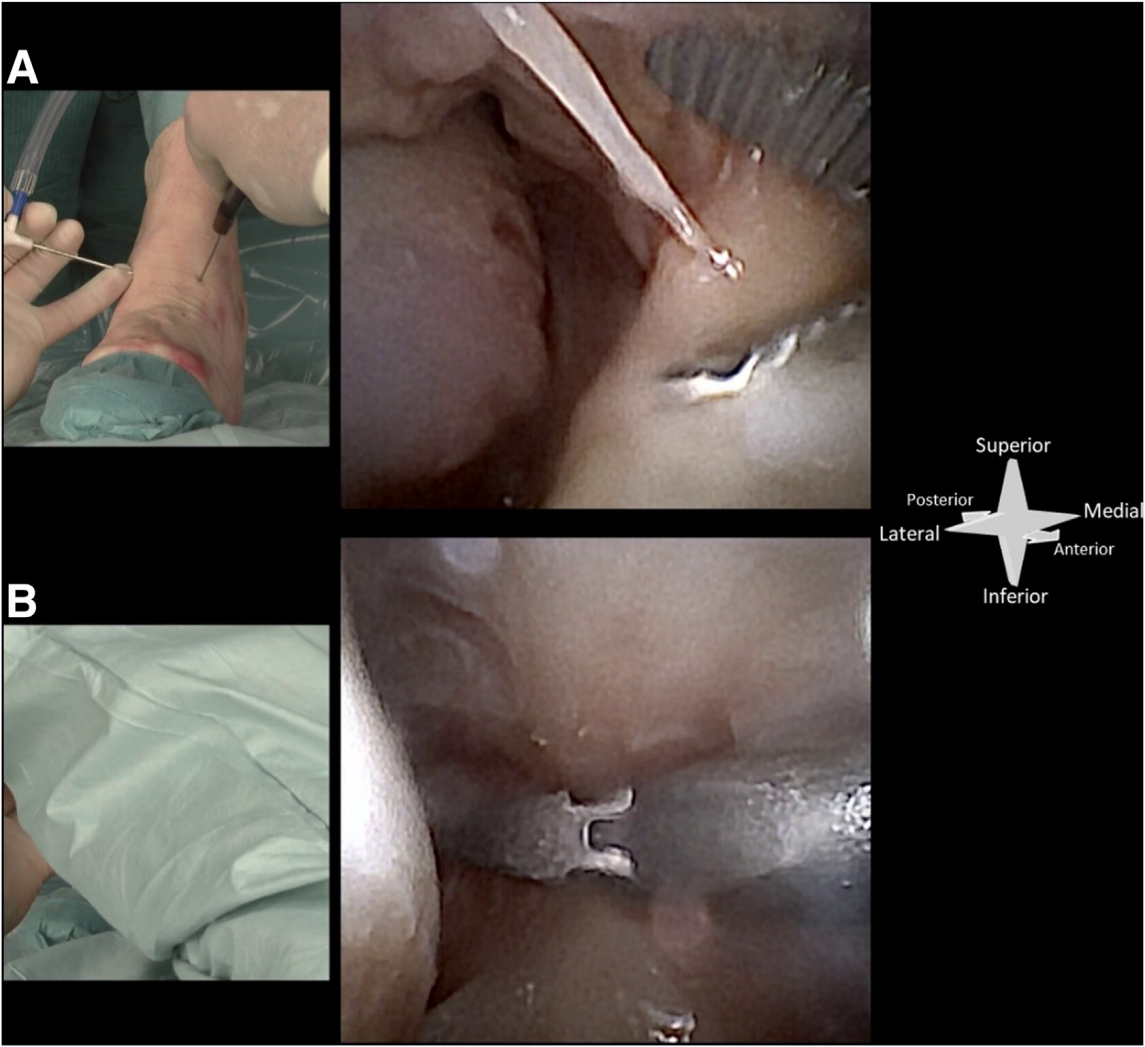
A left ankle in supine position seen from a top-down surgeon’s view. The needle arthroscope is inserted in the ankle joint through the anteromedial portal. A needle arthroscopic biter is inserted through the anterolateral portal and used to take synovial biopsies from the anterior (a) and more posterior (b) joint capsule.

Guided biopsies can be obtained from the joint capsule and synovium for histology or culture (Fig 6). A needle arthroscopic biter (NanoBiter; Arthrex) can be directly inserted through one of the portals. Note that instruments will generally not fit through the needle arthroscopic cannulas but should be inserted percutaneously through the portal.

### Debridement

**Fig 7:**
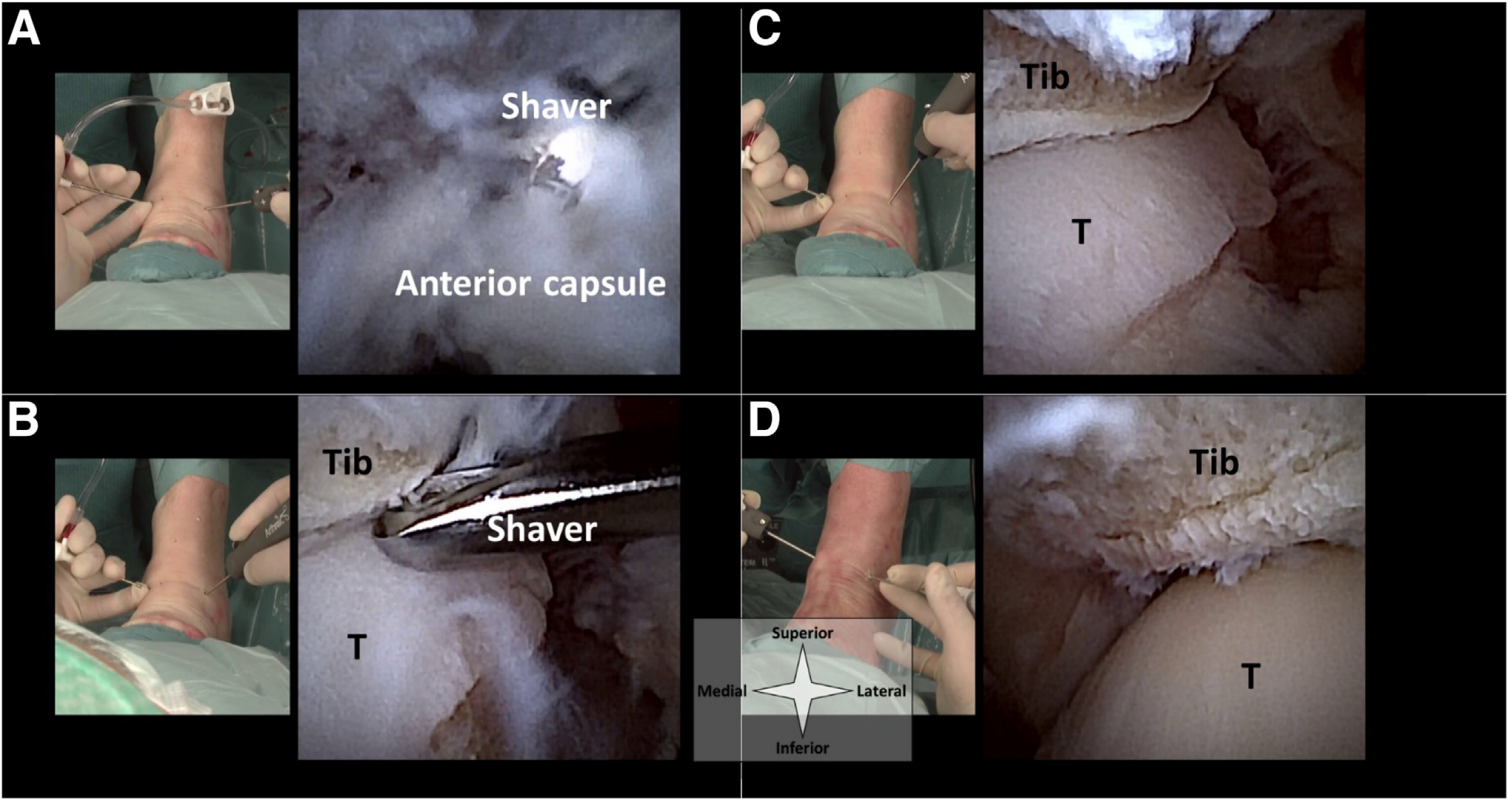
A left ankle in supine position seen from a top-down surgeon’s perspective, with corresponding intra-articular views from the needle arthroscope. A 2-mm diameter shaver is used to debride the proliferated anterior capsule (a) and bone spurs on the talar neck and distal tibia (b). The lateral (c) and medial (d) joint spaces are inspected for remaining potential for impingement by moving the ankle back and forth from plantarflexion to dorsiflexion. Note that the arthroscope and shaver are used in the anteromedial and anterolateral interchangeably. (T, talus, Tib, tibia.)

In case of bony or soft tissue impingement, 2-mm or 3-mm diameter shavers can be used for debridement (Fig 7). Hyperplastic synovium, cicatrized joint capsule, and osteophytes on the distal tibia rim and talar neck may be resected. With this small-diameter shaver equipment, it helps to connect a suctioning device to the shaver hand piece to maintain sufficient traction on tissue, and it should be noted that resection of a large amount of tissue will take more time compared to conventional shaver diameters.

### Delivery of Injectable Agents

**Fig 8:**
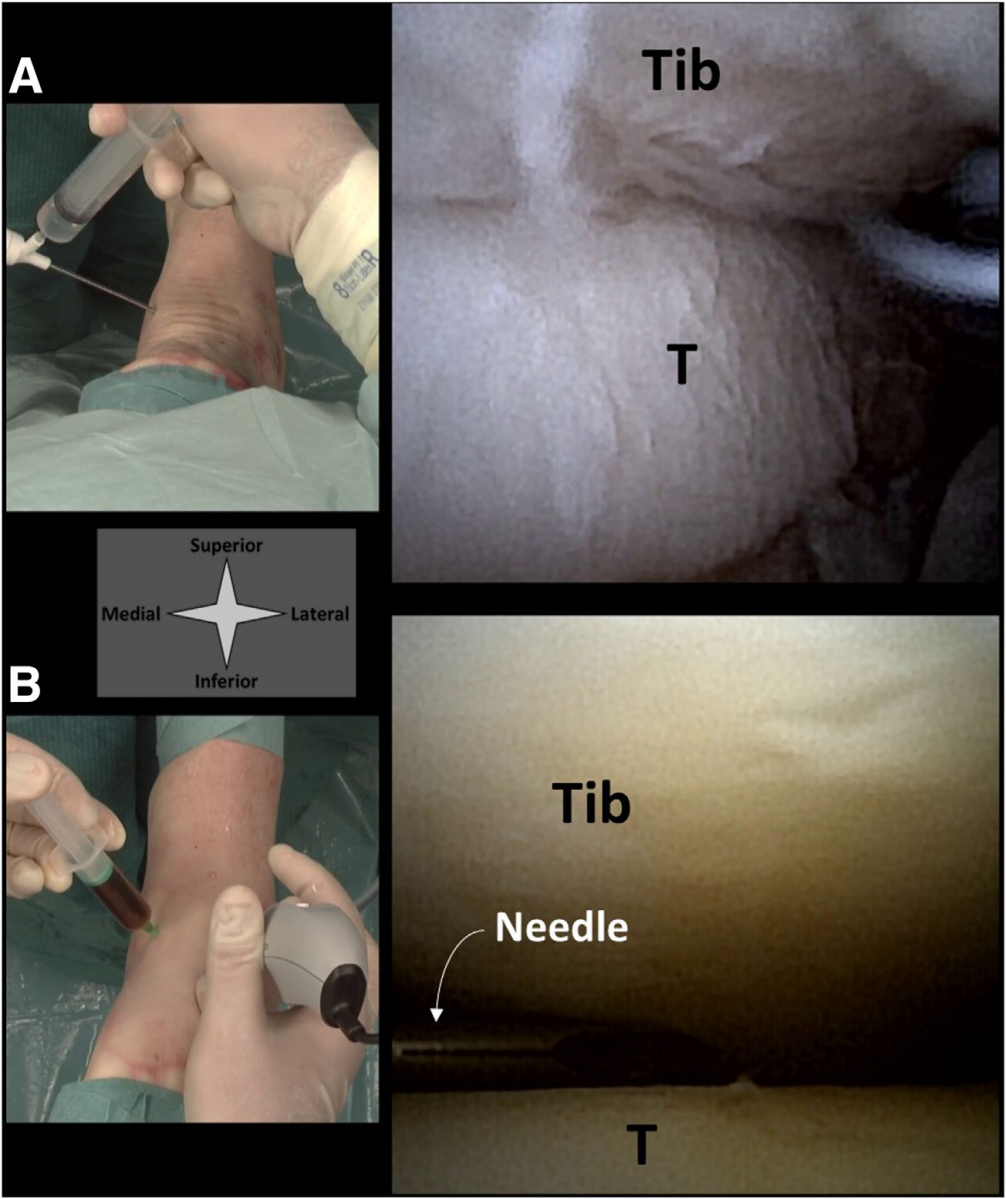
A left ankle in supine position seen from a top-down surgeon’s perspective, with corresponding intra-articular views from the needle arthroscope. Guided injections can be delivered either directly through the needle arthroscope with intra-articular positioning confirmed on the arthroscopic view (a) or by guiding a second syringe and needle to a specific location within the joint (b). (T, talus, Tib, tibia.)

Delivery of injectable agents can be performed in 2 ways (Fig 8). First, the injection can be delivered through the arthroscopic cannula itself. Once intra-articular positioning is confirmed with the arthroscopic view, a syringe can be directly connected to the cannula and the injectable agent can be delivered to the joint through the cannula. Second, needle arthroscopy can be used to confirm intra-articular placement of a second needle and syringe. This second needle can then be guided under direct visualization to specific areas or defects to deliver the injectable agent with precision.

### Closure

If needed, a final lavage can be performed to clear any debris. Subsequently, the joint is aspirated until it is completely dry, and all instruments are extracted. Since the procedure involves minimal soft tissue damage and uses small-diameter tools, sutures are not necessary. Instead, sterile wound closure strips or a simple band aid can be used to close the incisions, and if needed for comfort and additional hemostasis, a pressure bandage may be applied.

## Discussion

This Technical Note presents a standardized needle arthroscopic approach to the anterior ankle, with the possibility to obtain minimally invasive access to the joint for inspection and a variety of interventional options (e.g., debridement, nettoyage, biopsies, or injections). Although the concept of needle arthroscopy has been tried before, it was never broadly adopted due to inferior image quality and a lack of interventional possibilities.^5^ Recent innovation has increased image quality and made arthroscopic instruments with similarly small diameters available.^6^ This substantially improved the diagnostic capabilities of needle arthroscopy,^7^ and interventional options are arising alike.^8^, ^9^, ^10^, ^11^, ^12^, ^13^

Needle arthroscopy of the ankle has been found to be a safe procedure in cadaveric experiments,^2^ and needle arthroscopy in general has been reported to be associated with a low complication rate.^14^ Patient tolerability of needle arthroscopy of the ankle under local anesthesia has been tested as well and was found to be excellent in procedures ranging from inspection-injections to debridement of bony impingement and lavage for bacterial arthritis.^15^, ^16^, ^17^, ^18^

When considering needle arthroscopy for patients with ankle problems, it is important to consider potential pitfalls and disadvantages (Table 1). Introduction of the needle arthroscope, for example, has been shown to be excessively difficult in patients with end-stage osteoarthritis, and soft tissue that is cicatrized due to prior ankle surgery may hamper the procedure as well.^18^ In addition, patients should be counseled that a second conventional arthroscopic procedure may be required in the event of pathology that is more elaborate than was expected preoperatively. Nonetheless, needle arthroscopy offers several advantages. First, it enhances the overall patient experience and allows patients to observe the procedure if desired. Furthermore, it results in reduced soft tissue trauma, minimizing discomfort and promoting faster recovery. Additionally, this minimally invasive approach reduces the strain on valuable hospital resources like operating theaters and anesthesiology services, thereby helping to control hospital costs. These alleged merits will be scrutinized in further trials.

**Table 1:** Pearls and Pitfalls of Needle Arthroscopy of the Anterior Ankle

Pearls	Pitfalls
Procedures can be performed under local anesthesia and without sedation. Patients may be counseled with regard to their pathology intraoperatively, and the patient experience may be enhanced.	Arthroscope introduction may be excessively difficult in patients with complete ventral joint space obliteration in end-stage osteoarthritis or extensive soft tissue proliferation due to prior ankle surgery.
As general and spinal anesthesia are not required, there is less need for preoperative tests and screening, especially for patients with cardiovascular and pulmonary comorbidities.	In case of pathology that is more extensive than expected preoperatively, conversion may be needed to conventional arthroscopy or more aggressive anesthesia. Patients should be counseled for this possibility, which may necessitate planning of a second, separate procedure.
Positioning patients with their heels hanging just over the edge of the operating table will result in gravitational joint distention, which facilitates access to the joint. If needed, slight noninvasive distraction can be applied additionally.	If performed under local anesthesia, one should pay close attention to anesthesia of the joint capsule. Improper anesthesia of this tissue will result in a painful procedure.
Holding the needle arthroscope with a pencil grip will increase stability.	The 0° viewing angle requires a learning curve, as does the semi-flexibility of the arthroscope and instruments.
Introducing the arthroscopic cannula with a blunt obturator and the ankle in dorsiflexion will provide maximum protection of weightbearing cartilage.	The arthroscopic cannula is very thin, and there is room between the cannula and arthroscope. This poses a risk to making cartilage abrasions when approaching cartilage under a slope.

In conclusion, the technique presented here provides a standardized approach to needle arthroscopy of the anterior ankle, which may aid in safe, uniform, and beneficial adoption of this emergent technique.

## Disclosures

The authors report the following potential conflicts of interest or sources of funding: The Department of Orthopedic Surgery and Sports Medicine from the Amsterdam UMC was supported with an unrestricted research grant from Arthrex GmbH. G.M.M.J.K. received consultancy fees from Arthrex during the conduct of the study. S.A.S.S. serves as a board member for the Dutch Orthopedic Society. All other authors (T.S., A.B.W.) declare that they have no known competing financial interests or personal relationships that could have appeared to influence the work reported in this paper*.* Full ICMJE author disclosure forms are available for this article online, as supplementary material.

## Supplementary Data

ICMJE author disclosure forms

Video 1 A step-by-step demonstration of needle arthroscopy of the anterior ankle. For demonstration purposes, a cadaveric ankle specimen is used. A left ankle is seen from a top-down surgeon’s perspective, and corresponding needle arthroscopic views are shown where applicable. The video shows creation of the anteromedial portal first and the anterolateral portal second. The anteromedial portal is created at the medial soft spot. Be sure to keep the ankle in dorsiflexion during portal placement to protect the weightbearing cartilage. Needle arthroscopic inspection of the anterior ankle is shown. The needle arthroscope may be alternated between portals for proper inspection of the entire anterior joint. Subsequently, needle arthroscopic biters can be used to obtain soft tissue biopsies, shavers and burrs can be used for debridement of bony or soft tissue, and the procedure can be done to perform guided injections or lavage as well. Sutures are generally not required, and wound closure strips usually suffice for wound closure.
